# Exploration of the regulatory relationship between *KRAB-Zfp* clusters and their target transposable elements via a gene editing strategy at the cluster specific linker-associated sequences by CRISPR-Cas9

**DOI:** 10.1186/s13100-022-00279-x

**Published:** 2022-11-10

**Authors:** Yang Zhang, Fei He, Yanning Zhang, Qian Dai, Qintong Li, Jing Nan, Ruidong Miao, Bo Cheng

**Affiliations:** 1 School of Life Sciences, Lanzhou University, Key Laboratory of Cell Activities and Stress Adaptations, Ministry of Education, Lanzhou, Gansu People’s Republic of China 730000; 2 Departments of Obstetrics & Gynecology and Pediatrics, West China Second University Hospital, Key Laboratory of Birth Defects and Related Diseases of Women and Children, Ministry of Education, Development and Related Diseases of Women and Children Key Laboratory of Sichuan Province, Sichuan University, Chengdu, Sichuan People’s Republic of China 610041

**Keywords:** *KRAB-Zfp* cluster, Transposable elements, Linker sequence, CRISPR-Cas9

## Abstract

**Background:**

Krüppel Associated Box-containing Zinc Finger Proteins (KRAB-ZFPs), representing the largest superfamily of transcription factors in mammals, are predicted to primarily target and repress transposable elements (TEs). It is challenging to dissect the distinct functions of these transcription regulators due to their sequence similarity and diversity, and also the complicated repetitiveness of their targeting TE sequences.

**Results:**

Mouse *KRAB-Zfps* are mainly organized into clusters genomewide. In this study, we revealed that the intra-cluster members had a close evolutionary relationship, and a similar preference for zinc finger (ZnF) usage. *KRAB-Zfps* were expressed in a cell type- or tissue type specific manner and they tended to be actively transcribed together with other cluster members. Further sequence analyses pointed out the linker sequences in between ZnFs were conserved, and meanwhile had distinct cluster specificity. Based on these unique characteristics of *KRAB-Zfp* clusters, sgRNAs were designed to edit cluster-specific linkers to abolish the functions of the targeted cluster(s). Using mouse embryonic stem cells (mESC) as a model, we screened and obtained a series of sgRNAs targeting various highly expressed *KRAB-Zfp* clusters. The effectiveness of sgRNAs were verified in a reporter assay exclusively developed for multi-target sgRNAs and further confirmed by PCR-based analyses. Using mESC cell lines inducibly expressing Cas9 and these sgRNAs, we found that editing different *KRAB-Zfp* clusters resulted in the transcriptional changes of distinct categories of TEs.

**Conclusions:**

Collectively, the intrinsic sequence correlations of intra-cluster KRAB-Zfp members discovered in this study suggest that the conserved cluster specific linkers played crucial roles in diversifying the tandem ZnF array and the related target specificity of KRAB-Zfps during clusters’ evolution. On this basis, an effective CRISPR-Cas9 based approach against the linker sequences is developed and verified for rapidly editing *KRAB-Zfp* clusters to identify the regulatory correlation between the cluster members and their potential TE targets.

**Supplementary Information:**

The online version contains supplementary material available at 10.1186/s13100-022-00279-x.

## Introduction

KRAB-ZFP family represents the largest transcription factor superfamily in higher eukaryotes, with the number ranging from a few hundred to more than a thousand per species [1]. It has been experimentally proven that nearly 2/3 of the human KRAB-ZFPs target to TEs and are involved in the transcriptional repression [1, 2]. Being first traced back to the common ancestor of lungfish, coelacanth and tetrapod [1, 3, 4], KRAB-ZFPs are present in all vertebrates that have been studied [5], and presumably have undergone a rapid expansion upon the emergence of the new TEs [1, 6, 7]. Quite many of the KRAB-ZFPs remain the structural and functional similarities across species, and meanwhile each species has derived its unique sublineages of KRAB-ZFPs during evolution [2, 4, 8, 9].

A typical KRAB-ZFP protein is composed of an N-terminal KRAB domain and a C-terminal C2H2 ZnF array with an average of ~ 11 ZnFs in both mice and humans [10]. The conserved KRAB domains are responsible for recruiting Kap1/Trim28/Tif1β [11, 12], a core scaffold protein for assembling a variety of the epigenetic repressive complexes including histone H3 lysine 9 (H3K9) methyltransferase Setdb1 [13], histone deacetylase complexes NURD [14], DNA methyltransferases [15] etc. The DNA binding domains are composed of the C2H2 zinc finger arrays. The two cysteines and two histidines on each side of a ZnF chelating with one zinc ion to form a ββα structure and the amino acids located at the − 1, 3, 6 positions (according to the α-helix) usually mediate direct recognition for a DNA triplet, thus being designated as the “amino acid triplets” or the ZnF “fingerprint” [16–19].

To explore the functions of KRAB-ZFPs, the basic requirement is to identify their target genes/TEs. Due to the large number of KRAB-ZFPs and the complexity of their potential targets, testing them one by one experimentally via ChIP-Seq or other high throughput sequencing-based methods is no doubt a costly and daunting task [1, 6, 20, 21]. Currently, it is extremely challenging to predict the DNA-binding specificity (a combination of individual ZnFs) of a given KRAB-ZFP due to the following limitations [22, 23]. 1) For a certain ZnF, its preferably bound DNA triplets in live cells cannot be predicted accurately [24]. It has been found that besides those “fingerprint” amino acids, other nearby amino acids including the linker sequences also contribute to the DNA binding activity [17, 18, 25]. 2) It is commonly observed that not all the ZnF individuals within a tandem array are actually involved in the target recognition while the intrinsic rule of this selective usage is largely unknown [26]. 3) The sequence variations of the repetitive DNA elements make it difficult to accurately define the binding motifs for each KRAB-ZFP [21]. 4) The dynamic changes in the target sequences such as DNA methylation may also modulate the recognition by KRAB-ZFPs [27, 28].

Notably, the previous chromosomal distribution analyses uncovered that *KRAB-ZFPs* tended to be organized in clusters and share the effector domains [6, 8, 29–31], and a few studies demonstrated that some intra-cluster members were able to be co-expressed and target the same set of TEs [1, 6]. In this study, we mainly analyzed mouse *KRAB-Zfps* to further explore the significance and feasibility of studying these transcription factors in clusters. Through systematically comparing the intra-cluster *KRAB-Zfp* members, a series of their common features were captured, including the close evolutionary relationship, the similar preferences of the ZnF usages, and the partial co-expression with a cell type- or tissue type specificity. More interestingly, we found that the sequences of the linker regions were significantly more conserved than the internal ZnF sequences, especially among the intra-cluster *KRAB-Zfp* members, highlighting the potential function of these “fixed” flanking sequences in mediating the rearrangement of the ZnF units. Based on this finding, we designed and screened a series of sgRNAs efficiently targeting to the cluster-specific linker regions and achieved the cluster-wide gene editing via CRISPR-Cas9 technology using mouse ESC as a model. The results proved the feasibility and efficiency of this approach in the functional studies of *KRAB-Zfp* clusters.

## Results

### The KRAB-ZFP coding genes are primarily organized in clusters genomewide and the intra-cluster members tend to be evolutionarily close

**Fig. 1:**
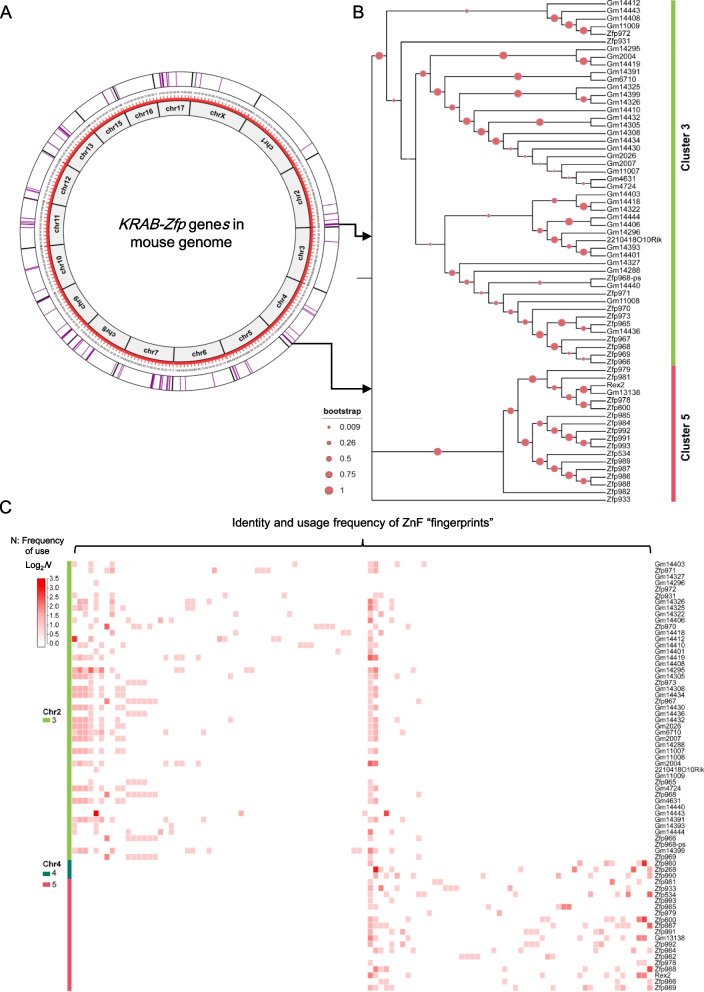
Analyses of the genomewide distribution, the evolutionary relationship, and the properties of ZnF usage in mouse *KRAB-Zfp* genes. **A** A circular genomic map displays the distribution of all *KRAB-Zfp* genes across the mouse genome. The width of each purple bar positively correlates to the number of *KRAB-Zfp* genes in a cluster. **B** A phylogenetic tree demonstrates the genetic distance among all the *KRAB-Zfp* genes located within *KRAB-Zfp* clusters 3 and 5. Bootstrap is a computer system function, representing the reliability of the branches of an evolutionary tree, ranging from 0 to 1. The larger the bootstrap value, the more reliable the branches of the evolutionary tree are. **C** Probability heatmap of the composition of ZnFs for KRAB-ZFPs in clusters 3, 4, and 5, including their identity and the frequency of occurrence. The identity of a ZnF is designated by its unique fingerprint (the amino acid triplets), see Fig. 3A for more details. Each square represents a unique type of ZnF/fingerprint and the intensity of the red color correlates to its frequency of occurrence in the corresponding KRAB-ZFP labeled on the right

It has been known that *KRAB-Zfp* genes in vertebrates tend to cluster in genome, including mice and humans [6, 29–32]. As the circular genomic map demonstrated in Fig. 1A, the mouse *KRAB-Zfps* (shown in purple lines) were commonly arranged next to each other to form clusters (the thick purple bars). For the convenience of reference, two parameters were used to artificially define the *KRAB-Zfp* clusters [10]: **①** The distance between two neighboring cluster members is no more than 200 kilobases; **②** The minimum number of *KRAB-Zfp* genes within one cluster is 2. Using these criteria, 50 *KRAB-Zfp* clusters were identified in the mouse genome, covering 302 out of the overall 357 *KRAB-Zfp* genes (Supplementary Table 1). Similarly, 46 *KRAB-ZFP* clusters were found in the human genome, covering 320 out of the total 380 genes, and more strikingly, more than half of the human *KRAB-ZFP* genes (216) were located on chromosome 19 (Supplementary Fig. 1 and Supplementary Table 1). Since the majority of *KRAB-Zfps* (~ 85% in both mice and humans) are located within clusters, this study is primarily focused on exploring the characteristics of the *KRAB-Zfp* clusters.

To compare the evolutionary relationship among KRAB-ZFPs within or across clusters, we constructed a full phylogenetic tree using the amino acid sequence information of all mouse KRAB-ZFPs (Supplementary Fig. 2). As indicated by the phylogenetic tree, the evolutionary relationship was significantly closer among the intra-cluster members compared with the cross-cluster members, although a small portion of the branches included the members from multiple clusters. Figure 1B demonstrates a simplified tree of two representative clusters (clusters 3 and 5), both belonging to the top 3 biggest clusters in mouse genome (48 members in cluster 3 and 18 in cluster 5, Supplementary Table 1) and being highly active in mESC. The results clearly demonstrated that KRAB-ZFP members in the same cluster were organized together and two branches were well separated from each other.

Since the DNA binding specificity of KRAB-ZFPs is determined by ZnFs, more concretely by their “fingerprints”, we next compared the sequence similarity of the ZnF fingerprints among the KRAB-ZFP members. The “fingerprint” sequences were extracted from all mouse KRAB-ZFP associated ZnFs (3741 ZnFs in total, carrying 1286 distinct types of the fingerprints) and each KRAB-ZFP was specified by both the identity and the occurrence frequency of its fingerprints (Supplementary Table 2). The observation as a whole sight indicated that the composition of fingerprints was quite cluster specific (Supplementary Fig. 3). As shown by the representative clusters, the patterns of fingerprint usage for cluster 3 and clusters 4/5 (clusters 4 and 5 are two neighboring clusters with similar features) were quite distinct from each other, while those for the intra-cluster members were actually very similar (Fig. 1C). Several fingerprints became the dominant species in certain cluster(s) due to their high frequency of usage. For example, there were 234 ZnFs in the clusters 4 and 5, and among them, 96 ZnFs (~ 41%) belonged to the top 5 fingerprint species that occurred at least 10 times in the cluster. Interestingly, many of these high frequently used ZnFs/fingerprints were found primarily or even exclusively within the particular cluster(s) throughout the genome (Supplementary Table 3). These findings strongly supported the hypothesis that the formation and expansion of *KRAB-Zfp* clusters mainly occurred locally in the genome.

### *KRAB-Zfp* cluster members exhibit the similar expression profiles in a cell type- or tissue specific manner

**Fig. 2:**
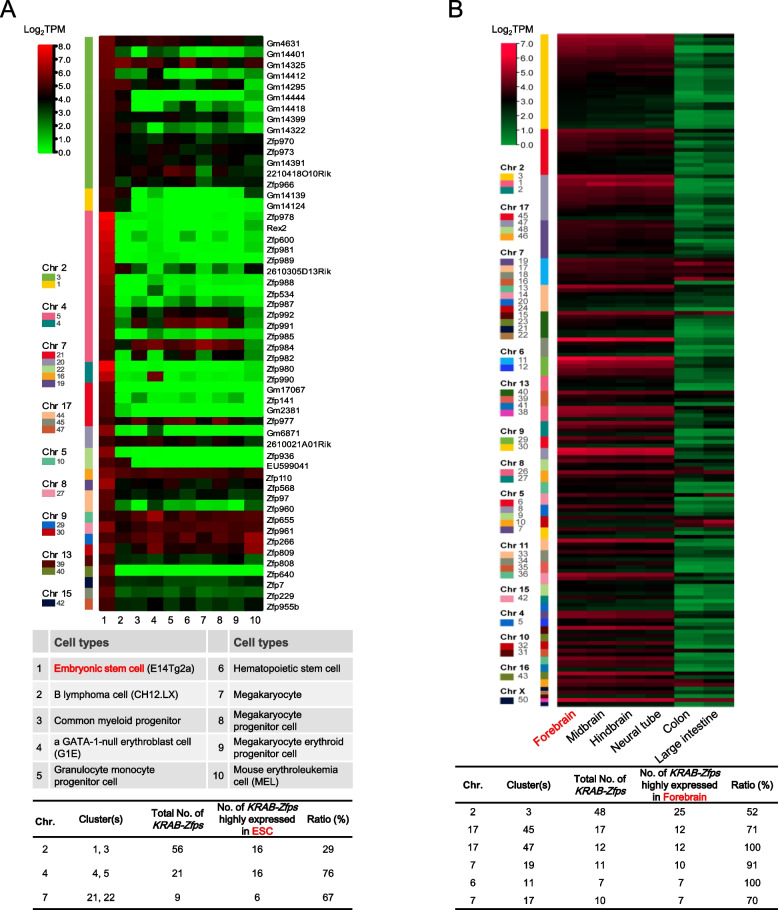
Analyses of the expression profiles of *KRAB-Zfp* clusters in various mouse cell lines and tissues. The heatmaps of the combined RNA-Seq data for the indicated cell lines (the detailed sample information is indicated in the table) and tissues are shown on the top. **A** The *KRAB-Zfps* highly expressed in mESC were selected (transcripts per million base pairs, TPM > = 25) and compared with the other cell types for their expression as indicated. **B** The *KRAB-Zfps* highly expressed in the forebrain were selected (TPM > = 4) and compared with the other indicated tissue types. For the main clusters highly expressed in mESC or in the forebrain, the ratios of the highly expressed *KRAB-Zfp* genes to the total number of *KRAB-Zfp* genes present in each cluster were calculated and shown in the tables at the bottom

Next, we performed the expression profiling analyses of *KRAB-Zfps* using the RNA-Seq data available in the literature [6]. *KRAB-Zfps* highly expressed in mESC were extracted and compared with the expression data of various cell types (Supplementary Table 4). As seen in the heatmap of Fig. 2A, the *KRAB-Zfps* highly expressed in mESC are mainly located within a few of the *KRAB-Zfp* clusters, including clusters 1, 3, and clusters 4, 5, etc., accounting for the large proportions of their whole gene clusters (~ 29–76%). Similar expression patterns are also seen in the other published datasets [32]. Some of the *KRAB-Zfps* are expressed stringently in mESC while others might express in the other cell types as well. Similarly, we provided an example to demonstrate the tissue specific expression of *KRAB-Zfps*. The expression data from four brain related tissues were used and compared to those of two digestive tract related tissues. As shown in Fig. 2B, quite many highly expressed *KRAB-Zfp* clusters (more than half of the cluster members were expressed) were identified in the brain related tissues, and in contrast, the expression of these genes was usually low in the digestive tract related tissues (Supplementary Table 4). These findings suggest that the expression of *KRAB-Zfp*s in the same cluster might be co-regulated. Therefore, manipulating gene expression of a *KRAB-Zfp* cluster can be developed as an efficient way to uncover its functions as a whole.

### The DNA sequences of the linker regions between ZnFs are conserved, especially within *KRAB-Zfp* clusters

The above findings suggest that it is valuable to explore the complicated functions of *KRAB-Zfp*s clusters. CRISPR-Cas9 based gene editing technology would be a very efficient and feasible strategy to achieve *KRAB-Zfp* cluster-knockout. *Wolf et. al* designed several sgRNA pairs on both sides of the *KRAB-Zfp* clusters and achieved a complete ablation of the target clusters [6]. Based on our findings, CRISPR-Cas9 might be executed more effectively via targeting the high frequently used cluster specific sequences. In order to identify such cluster specific sequences, we systematically carried out the following sequence analyses.

**Fig. 3:**
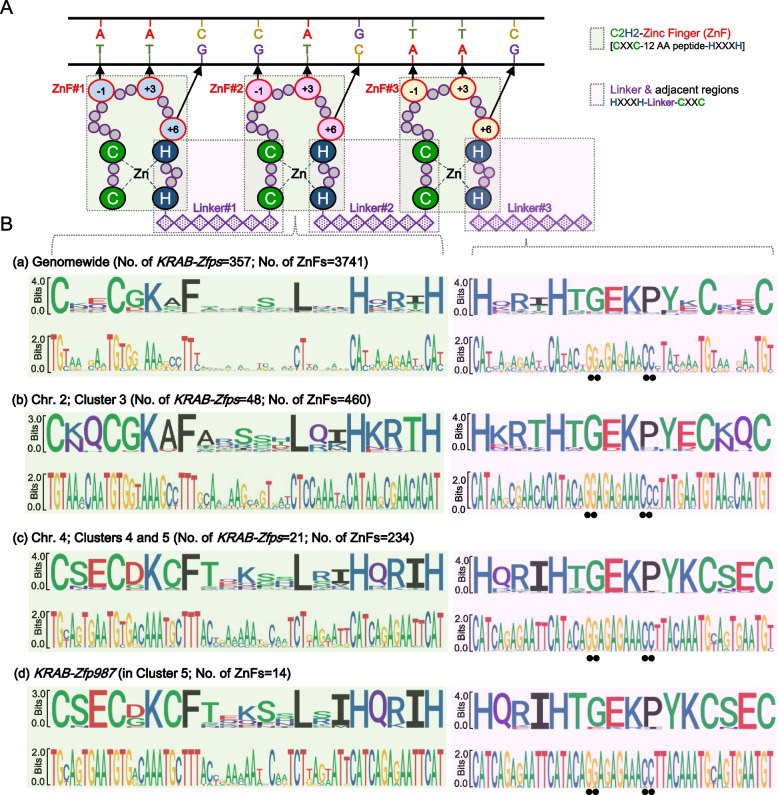
The sequence conservation analyses of the zinc finger domains of KRAB-ZFPs. (**A**) A schematic model of the interaction between a typical C2H2 zinc finger domain of KRAB-ZFP and its target DNA. (**B**) Sequence conservation analyses of the amino acid sequences (top) and the corresponding nucleic acid sequences (bottom) according to the regions shown in the bean green box or the pale purple box in (A). The related sequences were extracted from all the *KRAB-Zfps* across the mouse genome (**a**), within the *KRAB-Zfps* in cluster 3 (**b**), within the clusters 4 and 5 (**c**), or from one randomly selected *KRAB-Zfp* gene, namely *KRAB-Zfp987* (**d**)

Figure 3A is a sketch map of a simplified ZnF array [17, 18] and the entire region is fully covered by two sets of the overlapped “tiles”. One set is the whole ZnF region (designated as “ZnF”, including a fragment from CXXC to HXXXH, shaded in pale green) and the other set includes the linker sequence in between ZnFs together with its neighboring sequences (designated as a “Linker”, from HXXXH to CXXC, shaded in pale pink). Their corresponding sequences were extracted and systematically analyzed at three levels, all the *KRAB-Zfps* genomewide, the subgroups of the particular *KRAB-Zfps* cluster-wide, and an individual *KRAB-Zfp* level. The sequence alignments were visualized in the sequence logo diagrams in Fig. 3B, demonstrating the conservation of every single position (either an amino acid or a nucleotide) at each level. The results clearly indicated that the linker regions were significantly more conserved than the “ZnF” regions in all the three levels of analyses. The linker sequences were modestly conserved across all the clusters genomewide, while at a single cluster level, these sequences became fairly conserved. The SeqLog of all the KRAB-Zfps cluster members (302 genes) seems to be very similar to that of the overall 357 genes’, and the SeqLog analyses of 7 clusters are provided for comparison (Supplemental Fig. 2). More importantly, when making comparisons across the clusters, such as between cluster 3 and clusters 4/5 (the SeqLogs for cluster 4 and 5 are nearly identical), several conserved nucleotide variations were found, which endowed these linkers with a typical feature of cluster specificity. In addition, in a given *KRAB-Zfp*, these linker sequences are almost identical. Furthermore, we found the native DNA sequences in the linker regions contained two conserved “NGG” sites potentially used as PAM sites for sgRNA recognition (the black dots highlighted in the right panel of Fig. 3B and Supplementary Fig. 4). Taken together, we propose the linker sequences may be used as ideal sgRNA candidates for efficient editing of the *KRAB-Zfp* clusters.

### CRISPR-based genomic editing against the cluster-specific linker sequences revealed the targeting diversity of TEs by distinct KRAB-ZFP clusters in mESC

**Fig. 4:**
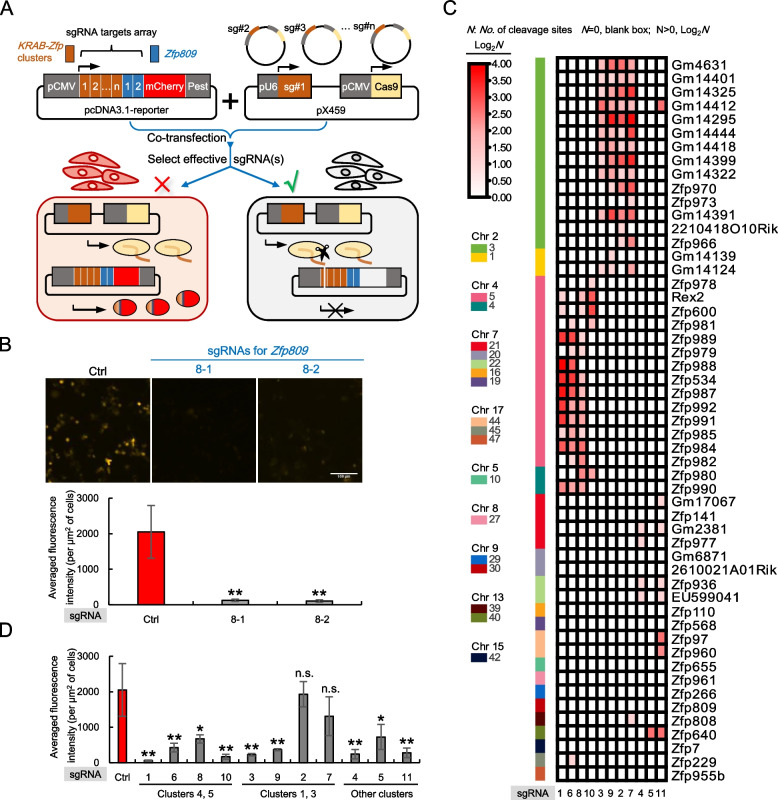
Screening effective sgRNAs for CRISPR-Cas9 mediated knockout of the *KRAB-Zfp* gene cluster(s). **A** A diagram illustrates the methodology of a CRISPR-Cas9 based reporter assay for screening the effective sgRNAs with multi-targets. SgRNAs that target to the cluster-specific linker sequences of *KRAB-Zfps* were designed and cloned into pX459, respectively. Their targeting sequences (including the corresponding PAM sites) were arranged in an array and fused together with the mCherry encoding sequences (PEST was added to accelerate the turn-over rate of the mCherry protein) and the entire open reading frame was ligated into the mammalian expression vector, pcDNA3.1. **B** Testing the screening assays in (A) with an empty pX459 vector (a negative control) and two positive sgRNA controls against *Zfp809.* The representative fluorescence image for each transfection was shown on top. The images were pseudo-colored to facilitate the recognition of some special readers. The bar graph at the bottom demonstrates the measured fluorescence changes of mCherry upon the expression of Cas9 and the indicated positive control sgRNAs. **C** A diagram demonstrates the target specificity and the numbers of the predicted cleavage sites of the sgRNAs specifically designed for the indicated *KRAB-Zfp* cluster(s). The color intensity is proportional to the number of the predicted cleavage sites. **D** The fluorescence changes of the mCherry reporter upon the expression of Cas9 together with the indicated sgRNAs listed in (C). The statistical analyses were carried out to compare each sgRNA’s effect to a negative control with an empty pX459 plasmid being transfected. “**” stands for an extremely significant change (*p*-value is smaller than 0.01); “*” stands for a significant change (p-value is greater than 0.01 but smaller than 0.05); n.s. stands for no significant change (p-value is greater than 0.05)

To test the feasibility of the above idea, we chose mESC as a model for the functional examination of *KRAB-Zfp* clusters. The sgRNAs were designed based on the sequences of the *KRAB-Zfp* clusters that highly expressed in mESC shown in Fig. 2A. The sgRNAs with the most predicted cleavage sites were selected with the highest priority. Altogether, 11 sgRNAs were designed and each contained several unique nucleotides that determine its targeting specificity to the corresponding *KRAB-Zfp* cluster(s) (Supplementary Fig. 5A). Different from the regularly used sgRNA, which usually targets a single-copy complementary sequence, each of the above sgRNAs potentially has a large number of the matched target sites. Technically speaking, it is challenging to evaluate the working efficiency of such sgRNAs. Thus, we developed a reporter assay to specifically screen the multi-target sgRNAs with good performance. As shown in Fig. 4A, the targeting sequences for all the sgRNAs to be tested were stitched together with a few more positive control sgRNAs included (proven to be efficient, Supplementary Fig. 5B). A reporter plasmid was constructed expressing a fusion gene composed of this tandem array of the sgRNA targeting sequences in the same frame with an mCherry reporter. Each unit contained the 20 bp-sgRNA targeting sequences along with their downstream 3 bp-PAM sites, and all the joints in between two units were optimized to guarantee everything fits in the same ORF as mCherry. Next, the reporter plasmid and a pX459 plasmid containing one of the sgRNAs were co-transfected into cells, and the functional sgRNAs were screened based on the decrease of the mCherry signals. The performance of this reporter system was tested by the two positive controls, respectively. As shown in Fig. 4B, in the presence of the empty vector (the negative control), the transfected reporter plasmid was able to express the mCherry gene. When a positive control sgRNA (8–1 or 8–2) was further expressed, the intensity of mCherry fluorescence was dramatically reduced, which proved that our reporter system was successfully established (Fig. 4B).

The targeting specificity and the numbers of the predicted cut sites of all the 11 sgRNAs were analyzed and demonstrated in Fig. 4C. Each sgRNA was able to recognize many sites within its targeting cluster, and in most cases, it did not target to any other *KRAB-Zfp* members beyond the cluster boundary, supporting the functional specificity of using these sgRNAs in mESC. We then evaluated all the cluster-specific sgRNAs one by one and the acquired images were further quantified and shown in Figs. 4D and Supplementary Fig. 5C (the raw data are provided in Supplementary Table 5). From these screenings, we successfully identified several effective sgRNA candidates against the clusters of interest (#1, #10; #3, #9; #4, #11).

More stringently, we searched the predicted cut sites of these sgRNAs across all the 357 mouse *KRAB-Zfps* genes and further selected the sgRNAs with most of their cut sites located within the target clusters (Supplementary Table 6). Three effective and specific sgRNAs were eventually selected (sgRNA1 for the targeting clusters 4 and 5, sgRNA3 for the targeting clusters 1 and 3, and sgRNA4 for the targeting clusters 21 and 22).

**Fig. 5:**
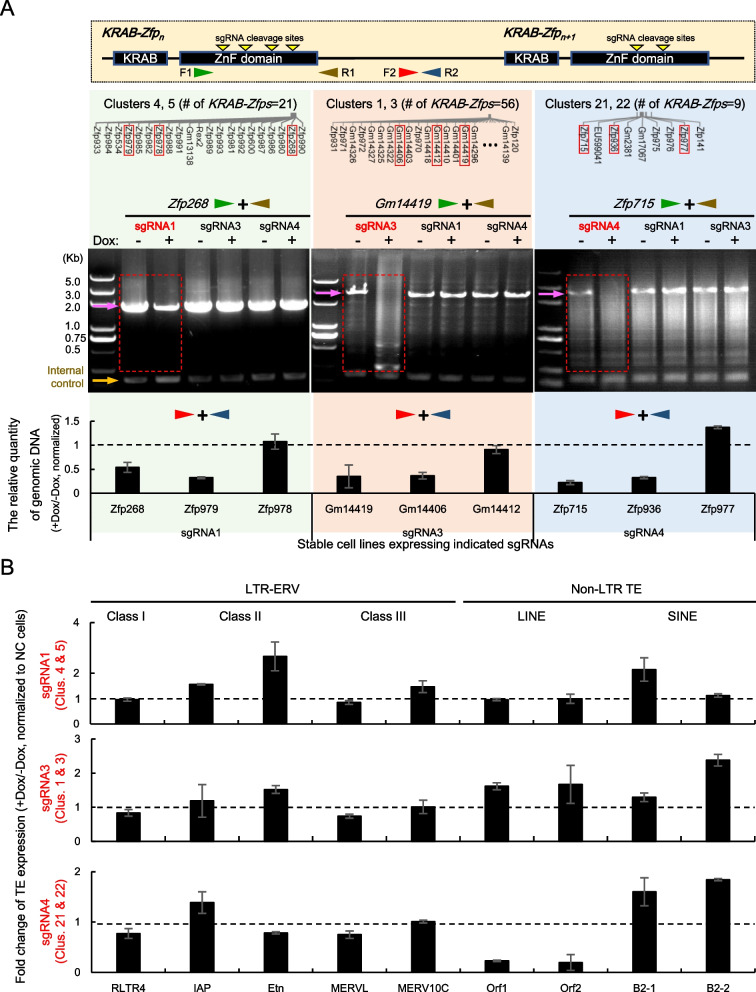
CRISPR-Cas9 mediated gene editing of *KRAB-Zfp* clusters and the effects on TE transcription in mESC. The mESC cell lines stably expressing the indicated sgRNA and inducibly expressing Cas9 were analyzed. **A** Verification of the efficiency and specificity of CRISPR-Cas9 mediated cleavage events in the targeted *KRAB-Zfp* clusters via PCR. Top panel: A Diagram of the primer design strategies. Primer pairs for directly detecting the zinc finger domains of the targeted *KRAB-Zfps* are indicated by F1 and R1 (the green and brown arrows) and the primers used to detect the intergenic regions of the two targeted *KRAB-Zfp* genes are indicated by F2 and R2 (the red and dark blue arrows). The bottom panel: The representative PCR or qPCR results for detecting the genomic DNA templates extracted before and after gene editing (−Dox and + Dox). The type of the primers is labeled on top using the arrow pairs with colors consistent with the diagram. For the specificity tests, the sgRNA labeled in red on top of the agarose gel images are the specific sgRNA designed for the *KRAB-Zfp* cluster of the genes tested, and the sgRNAs in black are sgRNAs designed for some other non-target clusters. The PCR products amplified from the original, non-edited control samples are indicated by pink arrows. An irrelevant DNA fragment was amplified in parallel and used as an internal control (the yellow arrow) for checking the quality and quantity of the templates used in the PCR reactions. The layout of the *KRAB-Zfp* genes located within the tested clusters are also shown on the top of the results and the ones with the effective PCR data are highlighted in the red boxes. For qPCR results shown at the bottom, the amplicons were located at the downstream intergenic regions of the indicated *KRAB-Zfp* genes. **B** RT-qPCR detection of the relative expression levels of the indicated classes of the repetitive DNA elements in mESC before and after gene editing (−Dox and + Dox) triggered by the sgRNAs labeled on the left. The qPCR data of the indicated TEs were normalized to the data of the *Gapdh* transcript, and the normalized data of −/+Dox samples of the sgRNA expressing mESC were further normalized to the data generated from the control cell line expressing the empty vector without any relevant sgRNA

Next, the effective sgRNAs were constructed into an expression vector respectively and transfected into a mESC cell line inducibly expressing Cas9 (Supplementary Fig. 5D) and the stable cell lines were screened. The cells expressing sgRNAs against distinct *KRAB-Zfp* clusters were treated with doxycycline for 3 days to induce Cas9 expression and the resultant gene editing events at the target loci. The gene editing effects were verified by PCR assays using the genomic DNA samples as the templates. To minimize the interference of the massive repetitive sequences in *KRAB-Zfps*, the specific primer pairs were designed either in the flanking regions of the zinc finger domain to amplify the entire zinc finger domain of a target *KRAB-Zfp*, or in the intergenic regions between two neighboring *KRAB-Zfps* to monitor the fragment loss caused by the multiple cleavage events. As shown in Fig. 5A (the agarose gel images), the PCR products of three randomly selected sgRNA-targeting *KRAB-Zfp* genes were significantly declined upon the expression of Cas9 and the matched sgRNAs, while the several non-targeting *KRAB-Zfp* genes did not show any change. These results directly proved that the efficient and specific cleavage events indeed occurred within the target *KRAB-Zfp* clusters. In addition, the qPCR results suggested that many of the genomic regions containing the tested fragments might get lost or rearranged to some extent upon the actions of the CRISPR machinery (Fig. 5A, the bottom bar graphs). Interestingly, the amplification signals for a few intergenic fragments did not get reduced upon the occurrence of gene editing (such as *Zfp978* etc.), implying that the neighboring sequences or other unknown factors might contribute to this preference of the sgRNA-mediated cleavage. Again, no significant expression changes were detected when checking the non-target *KRAB-Zfp* genes (Supplementary Fig. 6). Overall, the above results directly demonstrated the effectiveness and specificity of the tested sgRNAs in mediating Cas9 to cleave the target genes.

Based on the above results, RT-qPCR assays were carried out to analyze the expression changes of TEs upon the edition of the *KRAB-Zfp* clusters. The advantage of carrying out the inducible Cas9 expression system was that it allowed us to monitor the expression changes more accurately and timely. The results demonstrated that the disruption of different *KRAB-Zfp* clusters affected the expression of distinct collections of the repetitive DNA elements (Fig. 5B). For example, disruption of the clusters 4 and 5 by sgRNA1 resulted in the de-repression of several classes of LTR-ERVs including ETn, IAP, and disruption of the clusters 1 and 3 by sgRNA3 triggered the upregulation of the LINE elements, which were largely consistent to the results published in Wolf’s paper but the changes in our results seemed to be milder [6]. Interestingly, disruption of the clusters 21, and 22 by sgRNA 4 not only resulted in the increased expression of the B2 repeats of SINE, but also caused a dramatic reduction of the LINE expression. Taken together, these results uncovered the target specificity and unique functions of the mESC highly expressed *KRAB-Zfp* clusters, and supported the effectiveness and further applications of the CRISPR-Cas9 based linker-targeting strategy developed in this study.

## Discussion

KRAB-ZFPs play essential roles in mediating the transcriptional repression of TEs [1, 21, 33]. Currently, the accuracy of predicting KRAB-ZFPs’ targets is not satisfying, which strongly limits the exploration of their unique functions and the law of evolution [5, 34, 35]. *KRAB-Zfps* are primarily organized into gene clusters. In this study, we explored several intrinsic properties of *KRAB-Zfp* clusters. Phylogenetic analyses of all the 357 mouse KRAB-ZFPs revealed that the intra-cluster members had closer evolutionary relationship than the members in different clusters. The systematic analyses of all the 3741 mouse KRAB-ZFP associated ZnFs and their DNA-binding fingerprints uncovered that the intra-cluster members shared preference for the fingerprint usage. This phenomenon is very typical but not unique in mice, and similar observation was also obtained from the analyses of human KRAB-ZFPs on chromosome 19 (including 58% of the human KRAB-ZFPs) (Supplementary Fig. 7 and Supplementary Table 8). Consistent with these findings, the results of the sequence analyses further uncovered that the linker regions between ZnFs were highly conserved with some cluster specific features.

The cluster-wide distribution of the nearly identical ZnF linkers suggests that these conserved elements may be the sequence basis for ZnF rearrangements within and/or among *KRAB-Zfps* during the clusters’ evolution, directly facilitating the diversification of the *KRAB-Zfps.* Herein, we provide a hypothetical model regarding the cluster formation, which brings more details on the basis of the current “arm race” model [31, 36–39]. It is briefly described as followed. Initially, there was a few of *KRAB-Zfps* scattered in the genome. The emergence of new transposable DNA elements (TEs) broke the existing defense line of the host genome since they were free from the supervision of KRAB-ZFP-mediated epigenetic inhibition. The lasting retrotransposition activities of these new invaders (and possibly other cellular stresses) triggered genome instability and the accumulated evolving selective pressures resulted in the gene amplification of *KRAB-Zfps* in order to adapt to the changing of the TE loads in the “host” genome. Along with the rapid expansion of clusters, the conserved linkers-mediated massive DNA rearrangement events would occur, primarily locally and occasionally across clusters, such as deletion, insertion, or exchange of one or multiple ZnFs. During these processes, the target specificity of *KRAB-Zfps* was dynamically screened and specialized, and the “peace” of the genome would be reset until the emergence of the functionally matched defenders. Some evidence has been provided to support the key regulatory roles of the linkers as DNA recognition determinants during the evolutionary selection of *KRAB-Zfps* [40, 41]. Therefore, more thorough investigations are necessary to further uncover the correlations between the sequence variations of linkers and the DNA binding capability of their adjacent ZnFs.

The fact that *KRAB-Zfps* are differentially expressed across the distinct spatiotemporal states suggests the highly active members play crucial roles under the corresponding circumstances [6, 10]. Due to the long-term accumulation of sub-functionalization, it is seldom to find such a cluster with the expression of all its members being switched on or off perfectly in concert. However, when analyzing the RNA-Seq data of *KRAB-Zfps*, we identified a number of the cell type- or tissue type specific clusters, with significant proportions of their members co-expressed. Based on the close “consanguinity” of these intra-cluster members, we reason that the functional examinations on such *KRAB-Zfp* clusters are valuable for clarifying their molecular functions, regulatory mechanisms, and evolution patterns.

Based on the above findings, we were inspired to target the conserved linkers to achieve the functional ablation of various *KRAB-Zfp* cluster(s). We searched the conserved linker regions and identified two very conserved native “NGG” motifs that may potentially be utilized as the PAM sites for directing sgRNA-mediated loading of the CRISPR apparatus, and a series of sgRNAs were designed to edit the highly expressed *KRAB-Zfp* clusters in mESC. The efficiency and specificity of sgRNAs were screened in a reporter assay and also directly verified in mESC via PCR analyses. The results indicated that the genic editing of distinct *KRAB-Zfp* clusters resulted in the transcriptional changes of distinct TE families, which was largely consistent to the published information [6, 10]. The decreased expression of LINE by editing clusters 21 and 22 was somewhat unexpected since it was reported that KRAB-ZFPs were also involved in the repression of certain LINE lineages [42]. However, recently it was reported that certain types of LINE and SINE were spatially segregated under the help of their transcripts, which was essential for the formation of the higher-order chromatin structures during the early embryogenesis [43]. Thus, our observation of the simultaneous increase of SINE and decrease of LINE upon the gene editing of clusters 21 and 22 implies that these related KRAB-ZFPs play a role in regulating the transcriptional balance between SINE and LINE elements. Overall, these data proved the feasibility of our strategy for the cluster editing in the functional mining of *KRAB-Zfps*.

In a recently published paper, Wolf et al. performed CRISPR-Cas9 mediated cluster deletion via two sgRNAs targeting the flanking regions of each *KRAB-Zfp* cluster [6]. Our strategy is to target the conserved linker sequences that are used by the cluster members with a high frequency. The advantages and disadvantages of these two approaches are listed as followed. 1) Deletion of the entire region results in a complete loss of functions for the targeted *KRAB-Zfps* while it also results in the knockout of other functional genes present within the target *KRAB-Zfp* cluster. Our approach facilitates the gene editing of the targeted *KRAB-Zfp* cluster members more directly and precisely. It generally won’t cause a complete ablation of those neighboring non-*KRAB-Zfp* genes, and therefore helps overcome or reduce the potential lethality or the side effects raised by “off-target” knockout of the essential genes in a *KRAB-Zfp* cluster. 2) A single linker-based sgRNA used in our approach generally has an enhanced cleavage efficiency due to its multi-target characteristics. For example, 8 out of the 11 sgRNAs tested experimentally in this study had more than 30 cleavage sites within the targeted clusters (Supplementary Table 6). In contrast, the efficiency of deleting a large fragment (> 1 Mb) via two sgRNAs is usually low since both sgRNAs are required to function efficiently. 3) In terms of the targeting specificity, an sgRNA with a unique cleavage site is generally more specific than a multi-target sgRNA used in our approach. While in our case, we find it is quite feasible to design “cluster-specific sgRNAs” based on the unique sequence variations across the various clusters. For example, the sgRNA3 used in this study has 64 predicted cleavage sites in the mouse genome, 100% of them are present in the target cluster(s) (Supplementary Table 6). In addition, we were able to design multiple such highly specific sgRNAs against the various target *KRAb-Zfp* clusters, including the top 3 big clusters in the mouse genome (cluster 3 with 48 members, cluster 40 with 28 members, and cluster 5 with 18 members). Please refer to the Supplementary Table 6 for more details of their target specificity evaluation. Furthermore, we analyzed a couple of clusters composed of the mouse *KRAB-Zfp* related Satellite DNA identified in Kauzlaric’s paper [32], which were not listed among the 357 *KRAB-Zfps* genes. It turned out that those *KRAB-Zfp* related Satellite DNA clusters also exhibited the same features as the *KRAB-Zfp* clusters we described above (Supplementary Fig. 8). Their linkers and the nearby sequences were very conserved. Altogether, we conclude that the intrinsic cluster-specific features within and right next to the linkers make them be distinguishable from others and give our CRISPR-based strategy a broad application prospect. 4) Using our approach, if any potent, cluster-associated effect on transcription of TEs is obtained via analyzing the edited cell pool, a mutant library can be further generated via a single clone screening to lower the heterogeneity of the CRISPR efficacy. Further analyses would facilitate the identification of a subset of *KRAB-Zfp* genes or even a single *KRAB-Zfp* gene that plays the major regulatory roles. 5) Comparing to the stable knockout of the target cluster(s), our inducible CRISPR system provides a more timely and accurate approach to monitor the expression changes of TEs. The effects of the sgRNA-guided Cas9 cleavages can be optimized via adjusting the doxycycline incubation time. The expression changes of TEs shown in Fig. 5B were generated from a set of 3-day Dox treated mESC samples. Although the relative expression changes detected were not very drastic, these data were reproducible and presumably more objective because some side effects raised from a long-term cell selection were exempted to some extent. 6) Our approach results in a great number of cleavage events within the targeted cluster and potentially trigger the occurrence of recombination events. Alternatively, CRISPRi (CRISPR interference) [44] will achieve the same sgRNA-mediated targeting without trigger any DNA breakage and the following recombination events. In addition, it may be a lot easier to verify the CRISPRi effects via testing the transcripts of the targeted *KRAB-Zfps*. Overall, our approach may execute specific genome editing at multi-genes within a target *KRAB-Zpf* cluster more objectively and precisely, and it has a good potential to be further extended or improved.

## Conclusions

Studying the functions of *KRAB-Zfps* is an underexplored area of biology, which is worthy of interest because of the increasingly recognized importance of these transcriptional regulators and their TE targets in the regulation of gene expression.

In this study, a number of interesting properties about *KRAB-Zfp* gene clusters were uncovered. Most notably, we found that the linker sequences in between ZnFs were highly conserved and cluster specific. Together with the phenomenon of similar preference for ZnF usage among the intra-cluster members, we proposed a model about the potential roles of these linkers in the functional diversification of *KRAB-Zfps* during cluster formation.

Based on the above findings, we developed a new approach to achieve a rapid and efficient edit of the target *KRAB-Zfp* clusters and validated its effectiveness in their TE target identification. Further applications of this new strategy would help accelerate the functional explorations of *KRAB-Zfp* clusters in various cellular physiological or pathological conditions of interest.

## Methods

### Chromosomal position exhibition of *KRAB-Zfps*

The chromosomal position analyses of the mouse and human *KRAB-Zfps* were depicted using the “Advanced Circos” function of TBtools [45]. Two sets of annotation files were prepared and uploaded, providing the chromosomal sizing information and the genomic localization information for *KRAB-Zfps*, respectively. The annotation files about the mouse and human chromosomal sizing information were downloaded from the following websites (https://hgdownload.soe.ucsc.edu/goldenPath/hg19/bigZips/hg19.chrom.sizes; https://hgdownload.soe.ucsc.edu/goldenPath/mm10/bigZips/mm10.chrom.sizes). The genome localization information of human and mouse *KRAB-Zfps* are available in Supplemental Table 1, which was acquired from a published paper [10]. All these display functions were found as plugins in TBtools [46].

### Phylogenetic analysis

The protein sequences of all the mouse KRAB-ZFPs were downloaded from Corsinotti’s paper [10]. The protein sequences of the selected or all KRAB-ZFPs were exported in Alignment explorer of MEGA7 by Fasta format txt files and aligned via the ClustalW function of MEGA7. The aligned files were used to generate the corresponding Phylogenetic Analysis files. Then, neighbor-joining method was used for the phylogenetic tree construction and the number of bootstrap replications was set to 1000 and all the other parameters were in default. The calculated bootstrap values (correlating to the reliability of the branches of an evolutionary tree) were displayed as red dots on the phylogenetic tree using the ITOL website (https://itol.embl.de/).

### Seqlogo and fingerprint analysis

The same protein sequences of KRAB-ZFPs were employed as described above and the corresponding DNA sequences were obtained by Ensembl genome browser (https://asia.ensembl.org/). The R stringr package (https://CRAN.R-project.org/ package=stringr) was employed to extract the protein and DNA sequences of the ZnF regions and the corresponding linker regions as shown in Fig. 3B and Supplementary Fig. 4 (str_extract_all function). The Seqlogo results were displayed using the ggseqlogo package (https://CRAN.R-project.org/package=ggseqlogo).

Similarly, the fingerprint sequences were extracted from the ZnF sequences and connected using the str_sub and the str_c functions in the R stringr package, respectively. The frequency of use for each distinct type of fingerprint in every KRAB-ZFP was counted using a R function. A table was generated and shown as Supplementary Table 2 with the detailed information of the identity and frequency of use for all the KRAB-ZFP associated ZnF fingerprints. The whole matrix was visualized as a heatmap using the HeatMap function of TBtools.

### RNA-Seq data analyses

The RNA-Seq data of mouse *KRAB-Zfps* in 6 mouse tissues and 10 cell types were directly obtained from the Wolf’s article, and the sequencing reads were mapped using Tophat to ensure each mappable read to be reported once [6]. A collection of *KRAB-Zfps* highly expressed in mESC were selected (TPM > = 25) and the expression information of these *KRAB-Zfps* in the other 9 cell types (indicated in Fig. 2A) were extracted. Similarly, a collection of *KRAB-Zfps* highly expressed in forebrain were selected (TPM > = 4) and the expression information of these *KRAB-Zfps* in the other 5 cell types (indicated in Fig. 2B) were extracted. The extracted data were analyzed by R DESeq2 package [34]. Heatmaps were generated using the HeatMap function of TBtools. Within the heatmaps, the *KRAB-Zfps* located in the same cluster were arranged together in a descending order based on their expression values in mESC or in forebrain, respectively.

### Designing strategy and plasmid construction of cluster specific sgRNAs

The *KRAB-Zfp* clusters highly expressed in mESC were considered as our targets. By analyzing their DNA sequences, two conserved PAM sites were found within their linker regions. We extracted all the 20-bp DNA sequences (all possible sgRNAs) ahead of each PAM and counted the frequency of each (R stringr package). Eleven sgRNAs with the highest frequency (predicted as cleavage sites for Cas9) were selected for further testing. The heatmap about the potential cleavage profile of all the selected sgRNAs against all the mESC highly expressed *KRAB-Zfps* was generated using the HeatMap function of TBtools. The array of sgRNA targets (including the PAM triplets) and the mCherry-PEST fragment were commercially synthesized by GENEWIZ (Suzhou, China) and TQgene (Lanzhou, China), respectively. The two fragments were ligated into the mammalian expression vector, pcDNA3.1. Each of the designed individual sgRNA was cloned into pX459 [47], respectively. Efficiency verified sgRNAs were further constructed into pLenti-sgRNA-mCherry (a gift from Dr. Haojian Zhang, Wuhan University).

### Cell culture, transfection, and the generation and treatments of stable cell lines

The mESC line, E14Tg2a, was originally purchased from ATCC and trained to be feeder free. Cells were seeded in the cell culture plates precoated with 0.5% gelatin in DMEM medium (Gibco, 12800–017) supplemented with 15% Fetal Calf Serum (Gemini Bio-products, 900–108), 2 mM L-glutamine (Sangon, g22043B), 0.1 mM non-essential amino acid (Gibco, 11140–035), 100 U/mL penicillin G sodium, and 100 μg/mL streptomycin sulfate,1000 U/mL of LIF (lab purified).

HEK293T cells were cultured in DMEM medium supplemented with 10% Newborn Calf Serum (Biological Industries, 04–102-1A), 100 U/mL penicillin G sodium, and 100 μg/mL streptomycin sulfate. Transfection was performed following the manufacturer’s instructions (Vazyme, T101–01).

An E14Tg2a background mESC stable cell line inducibly expressing Cas9 (pCW-Cas9, Addgene 50661 [48]) was previously generated in our lab. This cell line was used as a parental cell line for further introducing sgRNA. For viral packaging, HEK-293 T cells (one well of a 6 well plate) were transfected with 1 μg pMD2G, 2 μg pAX2, and 3 μg of pLenti-sgRNA-mCherry (assembled with an effective sgRNA selected from our reporter assay) and the medium was refreshed at 6 hours post transfection. The viral stock was harvested after 72 hours and further infected the E14Tg2a: pCW-Cas9 cells for 12 hrs. Cells were recovered for 1 day and further subjected for puromycin selection (0.5 μg/mL) for 5 days. To monitor the CRISPR effects and the expression changes upon *KRAB-Zfp* knockout, the above cell lines with the indicated sgRNAs being expressed were treated with or without Doxycycline (2.5 μg/mL) for 3 days before harvest.

### Cell imaging and data analyses

HEK-293 T were co-transfected with the mCherry reporter and a pX459 carrying a single sgRNA to be tested. Twelve hours post transfection, Hoechst 33342 (Beyotime, C1028) was added and incubated for 10 mins to stain the nuclei before imaging acquirement using Cytation5 (BioTek, USA). The images for the bright field, DAPI, and RFP channels were taken and data analyses were carried out using Gene 5 software. The entire area of all cells in a given field were determined and measured via DAPI signals and the total fluorescence intensity was directly measured by the software. Based on these parameters, we calculated the averaged fluorescence signal using the total fluorescence intensity divided by the entire area (the raw data and their processing are provided in Supplementary Table 5).

### Genomic DNA extraction, primer design, and PCR-based analyses of CRISPR effects

The mESC cells (2 × 10^5^-1 × 10^6^ cells/sample) not treated or treated with doxycycline for 3 days were harvested and each cell pellet was resuspended with 300 μL of extraction buffer (200 mM Tris-HCl, PH 7.5, 250 mM NaCl, 25 mM EDTA, 0.5% SDS), extracted with the equal volume of phenol-chloroform-isoamyl alcohol (25:24:1, PH 7.7–8.3), and further precipitated with isopropanol and washed with 75% ethanol. Each of the precipitated genomic DNA samples was eventually resuspended in 70 μL of ddH_2_O, and the concentration was measured and adjusted to 50 ng/μL.

Primer pairs were designed for detecting the genomic DNA fragments of the randomly selected target *KRAB-Zfp* genes within the indicated clusters shown in Fig. 5A. The detailed primer information is provided in Supplementary Table 7. Two types of primer pairs were designed. One type was to specifically amplify the zinc finger domains of a target gene by regular PCR reactions (the sizes of the amplicons were usually ranging from one to several kilo base pairs). The PCR parameters, such as the annealing temperature and the extension time etc., were optimized for each primer pair. A primer pair amplifying a single copy genomic region was used as an internal control to carry out PCR reactions in parallel for verification of the quality and quantity of DNA templates across samples. One hundred ng of template was used in each 20 μL PCR reaction. The *KRAB-Zfp* related PCR product and 5 μL of the PCR product from the internal control reaction using the identical template were mixed and loaded onto agarose gels for analyses.

The other type of primer pairs was designed to amplify the intergenic regions between two target *KRAB-Zfps* in real-time quantitative PCR (qPCR) reactions. 50 ng of template was used in each 10 μL qPCR reaction containing 2 × SYBR Green qPCR Master Mix (Bimake, B21702, LowROX). The reactions were run on a qPCR thermocycler (ABI, Q5). A primer pair detecting the genomic DNA region of *Gapdh* was used as internal control to normalize the Ct values between -Dox and + Dox samples from the same mESC cell line expressing an sgRNA targeting to a *KRAB-Zfp* cluster of interest. The changes of Ct values were further normalized to the data of the control mESC cell line expressing the empty vector of pX459.

### RNA extraction, reverse transcription and real-time qPCR

Total RNA was extracted by the MolPure Cell/Tissue Total RNA Kit (YEASEN, 19221ES50) and the 2 μg of the total RNA was treated with RNase-Free DNaseI to remove the contaminated genomic DNA following the manufacturer’s instructions (Promega, M6101). Then the RNA was subjected to reverse transcription via the 1st strand cDNA synthesis SuperMix (YEASEN, 11141ES60) and the resultant cDNA was diluted into a final volume of 200 μL. For RT-qPCR, 2 μL of the diluted cDNA was used as templates in a 10 μL reaction. qPCR reactions were performed as described above. Primers used for detecting various TEs are listed in Supplementary Table 7. Data normalization was carried out the same as described above for genotyping qPCR assays, except that the primer pair used as the internal control was designed to detect the CDS region of *Gapdh*.

## Supplementary Information

**Additional file 1: Supplementary Fig. 1.** A circular genomic map displays the distribution of all *KRAB-ZFP* genes across the human genome. The width of each pink bar positively correlates to the number of *KRAB-ZFP* genes in a cluster.

**Additional file 2 Supplementary Fig. 2.** A phylogenetic tree demonstrates the genetic distance among all mouse *KRAB-Zfp* genes. The detailed description is the same as that in Fig. 1B.

**Additional file 3 Supplementary Fig. 3.** The combined features about the zinc fingers (ZnFs) for all the mouse KRAB-ZFPs, including the composition specificity and frequency of occurrence. The detailed description is the same as that in Fig. 1C. Each row represents a single KRAB-ZFP.

**Additional file 4 Supplementary Fig. 4.** Sequence conservation analyses of various *KRAB-Zfp* clusters. The amino acid sequences (top) and the corresponding nucleic acid sequences (bottom) according to the linker and nearby regions are shown in the pale purple box. The related sequences are extracted from all the *KRAB-Zfps* arranged in the 50 clusters across the mouse genome (a), or the indicated clusters on chromosome 2 (Clusters 1, 2, 3) (b), and on chromosome 4 (Clusters 4 and 5) (c), on chromosome 7 (Cluster 21) (d), or on chromosome 13 (Cluster 40) (e). The two NGG motifs are highlighted in black dots.

**Additional file 5 Supplementary Fig. 5.** Screening efficient sgRNAs for knocking out *KRAB-Zfp* cluster(s) by CRISPR-Cas9. (A) The sequences and detailed information of the 11 linker-targeting sgRNAs constructed in the reporter plasmid in Fig. 4A. There are two “NGG” sites within the linker regions and they were used as PAM sites for the two sets of sgRNAs. The nucleotides identical across the whole set of sgRNA targets are labeled in black and the variable nucleotides are highlighted in red. (B) The sequencing results of the targeted region in *Zfp809* after being edited by the indicated sgRNAs. These two sgRNAs were used as positive controls in the initial set-up of the reporter system and the cells transfected with an empty pX459 vector were sequenced as the non-edited negative controls. (C) The representative fluorescent images of the cells transfected with pX459 plasmid containing the indicated sgRNA and the reporter plasmid (the negative control with an empty pX459 vector transfected is shown in Fig. 4B). The images were pseudo-colored to facilitate all readers to read. (D) WB verification of the mESC line inducibly expressing Cas9-Flag. This cell line was used as a parental cell line for further generating stable cell lines expressing sgRNAs in the experiments shown in Fig. 5.

**Additional file 6 Supplementary Fig. 6.** Specificity tests of sgRNAs in qPCR assays. qPCR results for detecting the intergenetic regions between non-target KRAB-*Zfps* using the genomic DNA templates extracted from the cells expressing the indicated sgRNA before and after gene editing (−Dox and + Dox). The set of data are correlated to Fig. 5B.

**Additional file 7 Supplementary Fig. 7.** ZnF fingerprints analyses for human KRAB-ZFPs on chr19. The combined features about the ZnF fingerprints for KRAB-ZFPs for all the human KRAB-ZFPs on chromosome 19. The detailed description is the same as that in Fig. 1C. Each row represents a single KRAB-ZFP.

**Additional file 8 Supplementary Fig. 8.** Analyses of *KRAB-Zfp* related Satellite DNA cluster identified in the Kauzlaric’s paper [32]. (A) Comparison of the cluster distribution of our work and the Kauzlaric’s paper. The clusters defined by both studies are pointed with red arrows; the clusters only defined by our work are pointed with blue arrows; and the clusters only defined by the Kauzlaric’s paper are pointed with green arrows, mainly including the *KRAB-Zfp* related Satellite DNA elements. (B) SeqLogs of two representative clusters in the Kauzlaric’s paper are generated to show the sequence conservation around the linker regions (in a red box) and the chromosomal positions of the Satellite DNA elements used for making the SeqLogs are listed in the table below (downloaded from the supplementary table in the Kauzlaric’s paper. List of the Supplementary Tables.

**Additional file 9 Supplementary Table 1.** All mouse and human *KRAB-ZFP* genes (information for Fig. 1 A and S1A)

**Additional file 10 Supplementary Table 2.** The identity and frequency of all types of fingerprints in mouse KRAB-ZFP-associated ZnFs (information for Supplementary Fig. 3).

**Additional file 11 Supplementary Table 3.** High frequency ZnFs in clusters 3–5 (additional information for Fig. 1C).

**Additional file 12 Supplementary Table 4.** Expression profile for mouse *KRAB-Zfps* in various types and tissue types (information for Fig. 2).

**Additional file 13 Supplementary Table 5.** sgRNA efficiency test in reporter system (raw data for Fig. 4B and D).

**Additional file 14 Supplementary Table 6.** Target distribution analyses for sgRNAs used in Fig. 4.

**Additional file 15 Supplementary Table 7.** Primers used in Fig. 5.

**Additional file 16 Supplementary Table 8.** Fingerprints of the 216 Human *KRAB-ZFPs* on chr19.

## Data Availability

The sequence information and published datasets used for analyses in this work are clearly clarified in the Methods session. All the plasmids and stable cell lines generated in this study are available for sharing.

## Abbreviations

KRAB-ZFP: Krüppel Associated Box-containing Zinc Finger Proteins
TE: Transposable Element
ERV: Endogenous Retrovirus
LTR: Long Terminal Repeat
CRISPR: Clustered Regularly Interspaced Palindromic Repeats
SgRNA: Small guide RNA
LINE: Long Interspersed Elements
SINE: Short Interspersed Nuclear Elements
