# Supplementary figures and images for: Exploration of the regulatory relationship between KRAB-Zfp clusters and their target transposable elements via a gene editing strategy at the cluster specific linker-associated sequences by CRISPR-Cas9
Source: Mob DNA. 2022 Nov 10;13:25. doi: 10.1186/s13100-022-00279-x (PMC9647903; doi:10.1186/s13100-022-00279-x)

## Slide 1
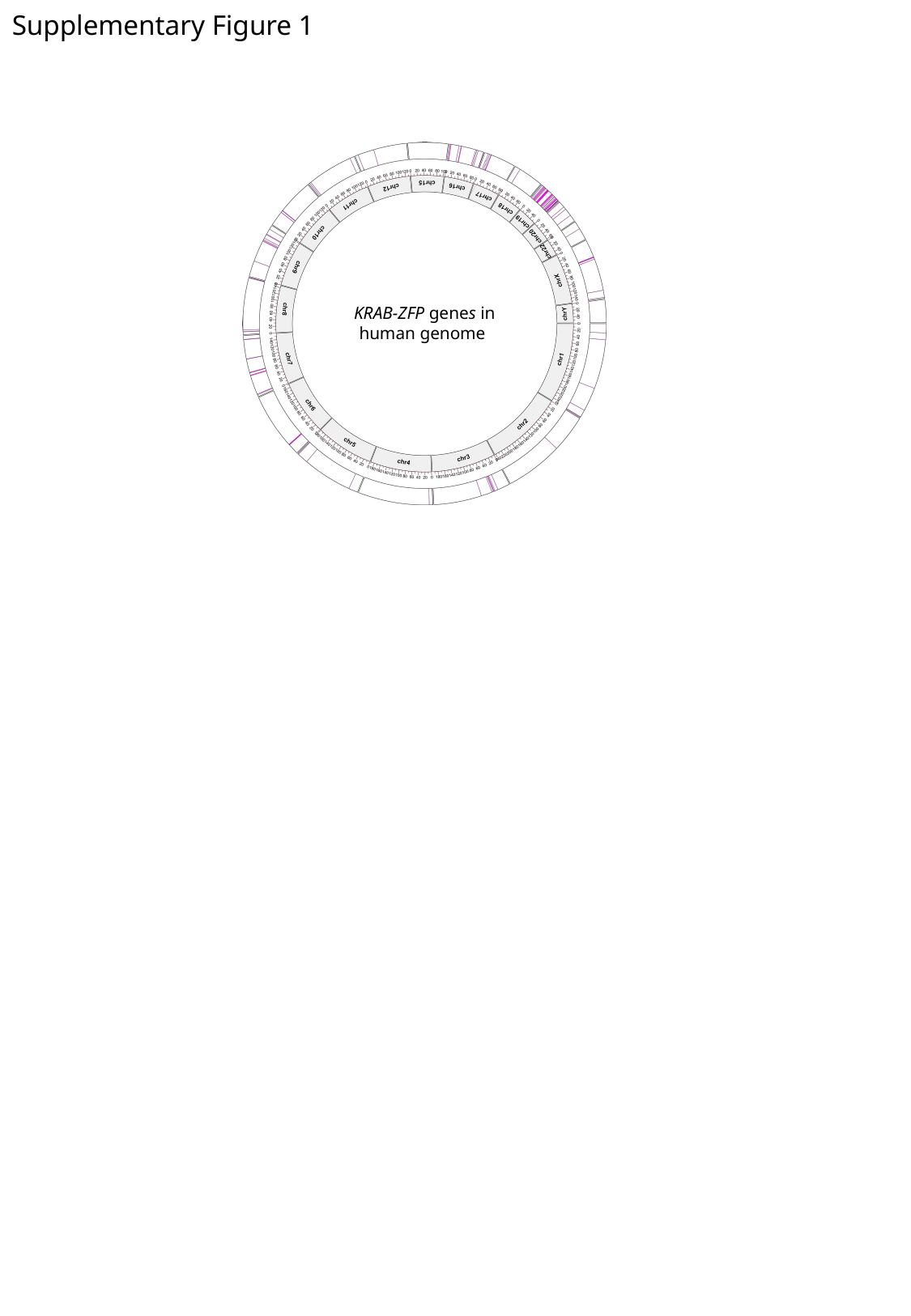

Supplementary Figure 1
KRAB-ZFP genes in human genome

Supplement: Supplementary file 1 — Additional file 1: Supplementary Fig. 1. A circular genomic map displays the distribution of all KRAB-ZFP genes across the human genome. The width of each pink bar positively correlates to the number of KRAB-ZFP genes in a cluster. [file 13100_2022_279_MOESM1_ESM.pptx]
