# Supplementary material for: Exploration of the regulatory relationship between KRAB-Zfp clusters and their target transposable elements via a gene editing strategy at the cluster specific linker-associated sequences by CRISPR-Cas9
Source: Mob DNA. 2022 Nov 10;13:25. doi: 10.1186/s13100-022-00279-x (PMC9647903; doi:10.1186/s13100-022-00279-x)
Supplement: Supplementary file 4 — Additional file 4 Supplementary Fig. 4. Sequence conservation analyses of various KRAB-Zfp clusters. The amino acid sequences (top) and the corresponding nucleic acid sequences (bottom) according to the linker and nearby regions are shown in the pale purple box. The related sequences are extracted from all the KRAB-Zfps arranged in the 50 clusters across the mouse genome (a), or the indicated clusters on chromosome 2 (Clusters 1, 2, 3) (b), and on chromosome 4 (Clusters 4 and 5) (c), on chromosome 7 (Cluster 21) (d), or on chromosome 13 (Cluster 40) (e). The two NGG motifs are highlighted in black dots. [file 13100_2022_279_MOESM4_ESM.pptx]

## Slide 1
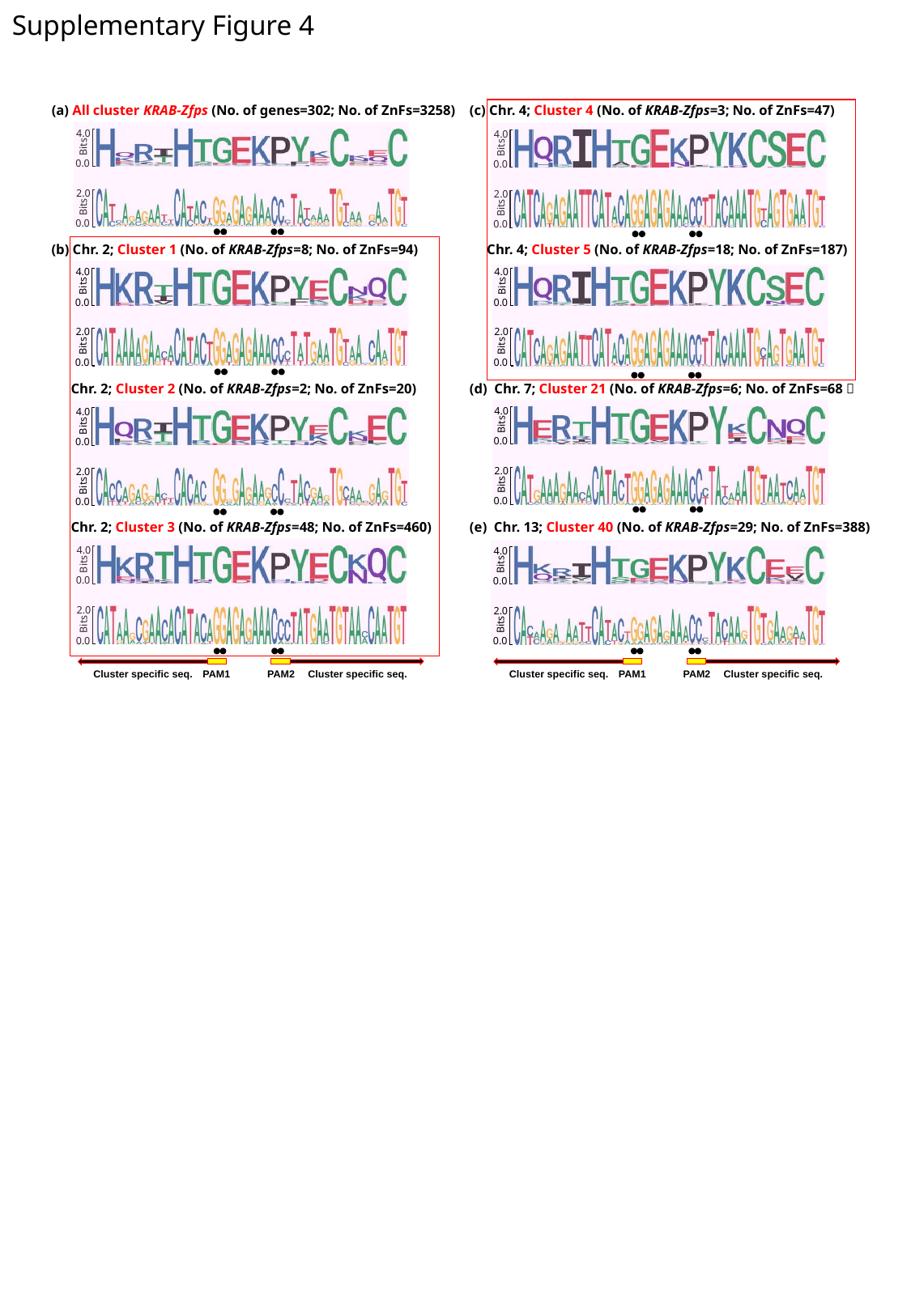

Supplementary Figure 4
(a) All cluster KRAB-Zfps (No. of genes=302; No. of ZnFs=3258)
(c) Chr. 4; Cluster 4 (No. of KRAB-Zfps=3; No. of ZnFs=47)
4.0
Bits
0.0
2.0
Bits
0.0
4.0
Bits
0.0
2.0
Bits
0.0
(b) Chr. 2; Cluster 1 (No. of KRAB-Zfps=8; No. of ZnFs=94)
 Chr. 4; Cluster 5 (No. of KRAB-Zfps=18; No. of ZnFs=187)
4.0
Bits
0.0
2.0
Bits
0.0
4.0
Bits
0.0
2.0
Bits
0.0
 Chr. 2; Cluster 2 (No. of KRAB-Zfps=2; No. of ZnFs=20)
(d) Chr. 7; Cluster 21 (No. of KRAB-Zfps=6; No. of ZnFs=68）
4.0
Bits
0.0
2.0
Bits
0.0
4.0
Bits
0.0
2.0
Bits
0.0
 Chr. 2; Cluster 3 (No. of KRAB-Zfps=48; No. of ZnFs=460)
(e) Chr. 13; Cluster 40 (No. of KRAB-Zfps=29; No. of ZnFs=388)
4.0
Bits
0.0
2.0
Bits
0.0
4.0
Bits
0.0
2.0
Bits
0.0
Cluster specific seq.
PAM1
PAM2
Cluster specific seq.
Cluster specific seq.
PAM1
PAM2
Cluster specific seq.
