# Supplementary material for: Exploration of the regulatory relationship between KRAB-Zfp clusters and their target transposable elements via a gene editing strategy at the cluster specific linker-associated sequences by CRISPR-Cas9
Source: Mob DNA. 2022 Nov 10;13:25. doi: 10.1186/s13100-022-00279-x (PMC9647903; doi:10.1186/s13100-022-00279-x)
Supplement: Supplementary file 5 — Additional file 5 Supplementary Fig. 5. Screening efficient sgRNAs for knocking out KRAB-Zfp cluster(s) by CRISPR-Cas9. (A) The sequences and detailed information of the 11 linker-targeting sgRNAs constructed in the reporter plasmid in Fig. 4A. There are two “NGG” sites within the linker regions and they were used as PAM sites for the two sets of sgRNAs. The nucleotides identical across the whole set of sgRNA targets are labeled in black and the variable nucleotides are highlighted in red. (B) The sequencing results of the targeted region in Zfp809 after being edited by the indicated sgRNAs. These two sgRNAs were used as positive controls in the initial set-up of the reporter system and the cells transfected with an empty pX459 vector were sequenced as the non-edited negative controls. (C) The representative fluorescent images of the cells transfected with pX459 plasmid containing the indicated sgRNA and the reporter plasmid (the negative control with an empty pX459 vector transfected is shown in Fig. 4B). The images were pseudo-colored to facilitate all readers to read. (D) WB verification of the mESC line inducibly expressing Cas9-Flag. This cell line was used as a parental cell line for further generating stable cell lines expressing sgRNAs in the experiments shown in Fig. 5. [file 13100_2022_279_MOESM5_ESM.pptx]

## Slide 1
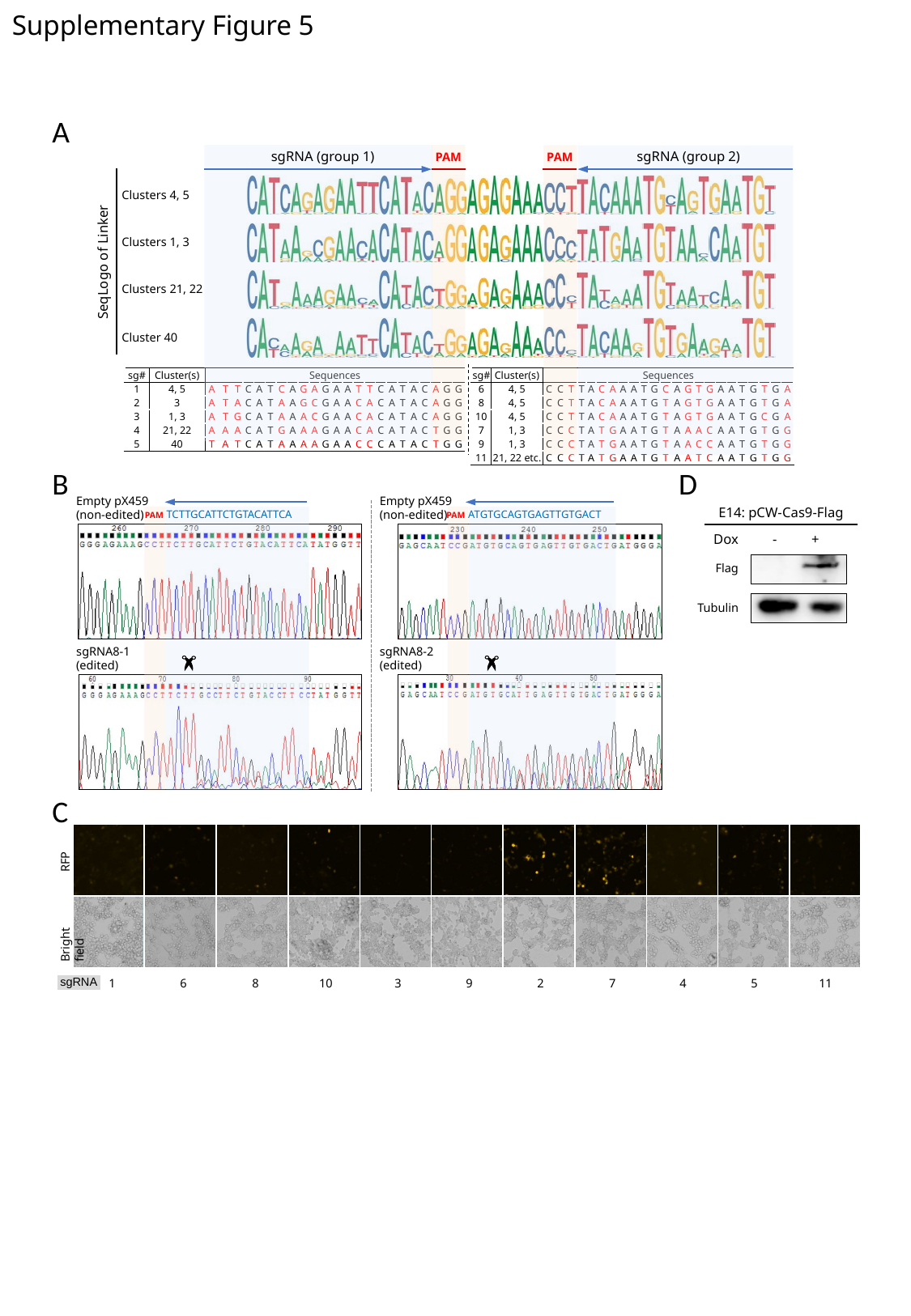

Supplementary Figure 5
A
sgRNA (group 1)
sgRNA (group 2)
PAM
PAM
Clusters 4, 5
Clusters 1, 3
SeqLogo of Linker
Clusters 21, 22
Cluster 40
| sg# | Cluster(s) | Sequences | | | | | | | | | | | | | | | | | | | | | | |
| --- | --- | --- | --- | --- | --- | --- | --- | --- | --- | --- | --- | --- | --- | --- | --- | --- | --- | --- | --- | --- | --- | --- | --- | --- |
| 1 | 4, 5 | A | T | T | C | A | T | C | A | G | A | G | A | A | T | T | C | A | T | A | C | A | G | G |
| 2 | 3 | A | T | A | C | A | T | A | A | G | C | G | A | A | C | A | C | A | T | A | C | A | G | G |
| 3 | 1, 3 | A | T | G | C | A | T | A | A | A | C | G | A | A | C | A | C | A | T | A | C | A | G | G |
| 4 | 21, 22 | A | A | A | C | A | T | G | A | A | A | G | A | A | C | A | C | A | T | A | C | T | G | G |
| 5 | 40 | T | A | T | C | A | T | A | A | A | A | G | A | A | C | C | C | A | T | A | C | T | G | G |
| sg# | Cluster(s) | Sequences | | | | | | | | | | | | | | | | | | | | | | |
| --- | --- | --- | --- | --- | --- | --- | --- | --- | --- | --- | --- | --- | --- | --- | --- | --- | --- | --- | --- | --- | --- | --- | --- | --- |
| 6 | 4, 5 | C | C | T | T | A | C | A | A | A | T | G | C | A | G | T | G | A | A | T | G | T | G | A |
| 8 | 4, 5 | C | C | T | T | A | C | A | A | A | T | G | T | A | G | T | G | A | A | T | G | T | G | A |
| 10 | 4, 5 | C | C | T | T | A | C | A | A | A | T | G | T | A | G | T | G | A | A | T | G | C | G | A |
| 7 | 1, 3 | C | C | C | T | A | T | G | A | A | T | G | T | A | A | A | C | A | A | T | G | T | G | G |
| 9 | 1, 3 | C | C | C | T | A | T | G | A | A | T | G | T | A | A | C | C | A | A | T | G | T | G | G |
| 11 | 21, 22 etc. | C | C | C | T | A | T | G | A | A | T | G | T | A | A | T | C | A | A | T | G | T | G | G |
B
D
Empty pX459
(non-edited)
Empty pX459
(non-edited)
E14: pCW-Cas9-Flag
PAM TCTTGCATTCTGTACATTCA
PAM ATGTGCAGTGAGTTGTGACT
Dox - +
Flag
Tubulin
sgRNA8-1 (edited)
sgRNA8-2 (edited)
C
RFP
Bright field
1
6
8
10
3
9
2
7
4
5
11
| sgRNA |
| --- |
