# Supplementary material for: Exploration of the regulatory relationship between KRAB-Zfp clusters and their target transposable elements via a gene editing strategy at the cluster specific linker-associated sequences by CRISPR-Cas9
Source: Mob DNA. 2022 Nov 10;13:25. doi: 10.1186/s13100-022-00279-x (PMC9647903; doi:10.1186/s13100-022-00279-x)
Supplement: Supplementary file 6 — Additional file 6 Supplementary Fig. 6. Specificity tests of sgRNAs in qPCR assays. qPCR results for detecting the intergenetic regions between non-target KRAB-Zfps using the genomic DNA templates extracted from the cells expressing the indicated sgRNA before and after gene editing (−Dox and + Dox). The set of data are correlated to Fig. 5B. [file 13100_2022_279_MOESM6_ESM.pptx]

## Slide 1
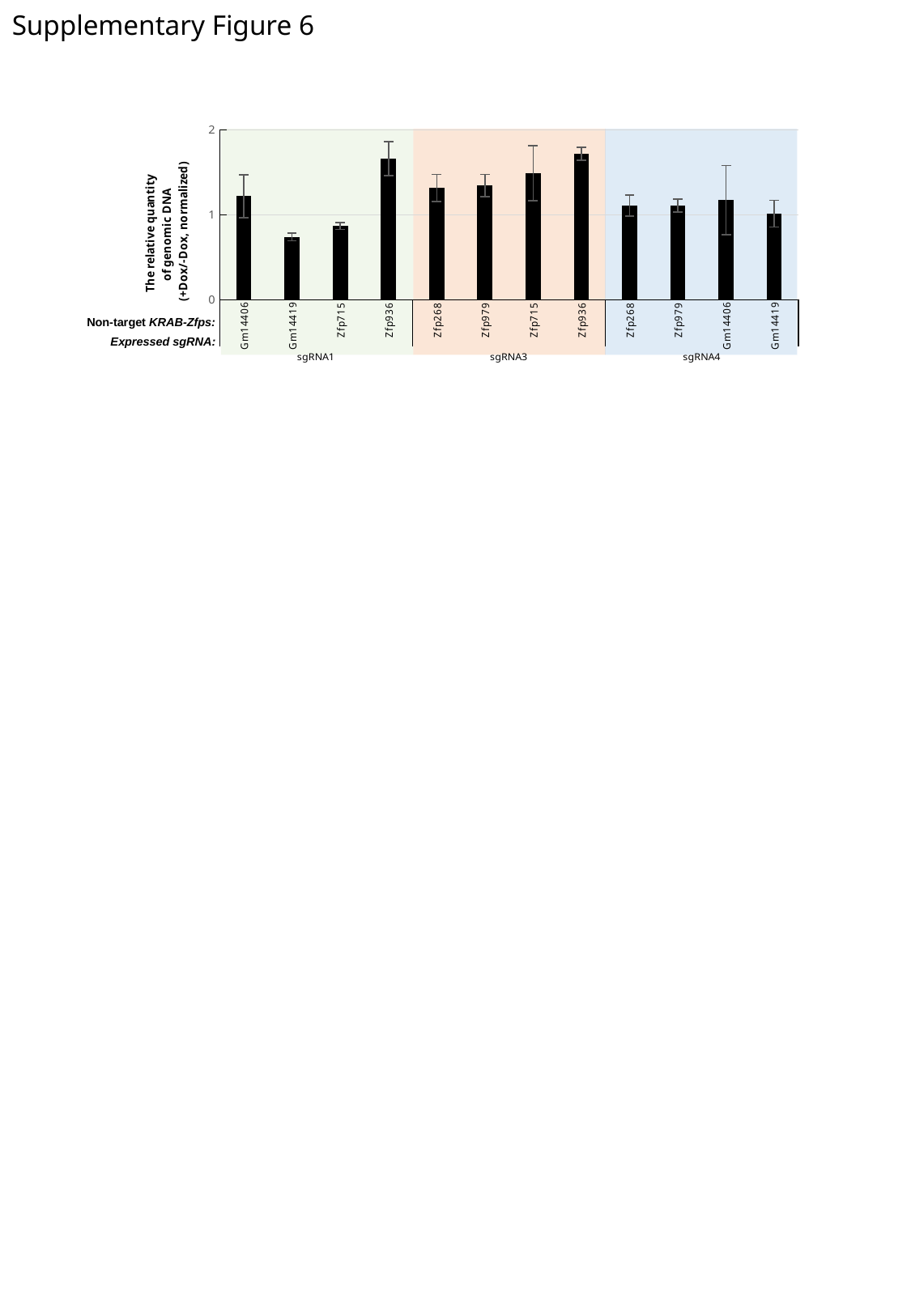

Supplementary Figure 6
### Chart
| Category | |
|---|---|
| Gm14406 | 1.216968258538168 |
| Gm14419 | 0.7379994560823228 |
| Zfp715 | 0.8666290478698092 |
| Zfp936 | 1.6608850887236994 |
| Zfp268 | 1.3134601130161219 |
| Zfp979 | 1.3439133615583168 |
| Zfp715 | 1.4865539136777346 |
| Zfp936 | 1.7159200711079523 |
| Zfp268 | 1.1081960163905804 |
| Zfp979 | 1.1082342956716897 |
| Gm14406 | 1.1713573480152513 |
| Gm14419 | 1.0109399349713992 |
Non-target KRAB-Zfps:
Expressed sgRNA:
