# Supplementary material for: Exploration of the regulatory relationship between KRAB-Zfp clusters and their target transposable elements via a gene editing strategy at the cluster specific linker-associated sequences by CRISPR-Cas9
Source: Mob DNA. 2022 Nov 10;13:25. doi: 10.1186/s13100-022-00279-x (PMC9647903; doi:10.1186/s13100-022-00279-x)
Supplement: Supplementary file 8 — Additional file 8 Supplementary Fig. 8. Analyses of KRAB-Zfp related Satellite DNA cluster identified in the Kauzlaric’s paper [32]. (A) Comparison of the cluster distribution of our work and the Kauzlaric’s paper. The clusters defined by both studies are pointed with red arrows; the clusters only defined by our work are pointed with blue arrows; and the clusters only defined by the Kauzlaric’s paper are pointed with green arrows, mainly including the KRAB-Zfp related Satellite DNA elements. (B) SeqLogs of two representative clusters in the Kauzlaric’s paper are generated to show the sequence conservation around the linker regions (in a red box) and the chromosomal positions of the Satellite DNA elements used for making the SeqLogs are listed in the table below (downloaded from the supplementary table in the Kauzlaric’s paper. List of the Supplementary Tables. [file 13100_2022_279_MOESM8_ESM.pptx]

## Slide 1
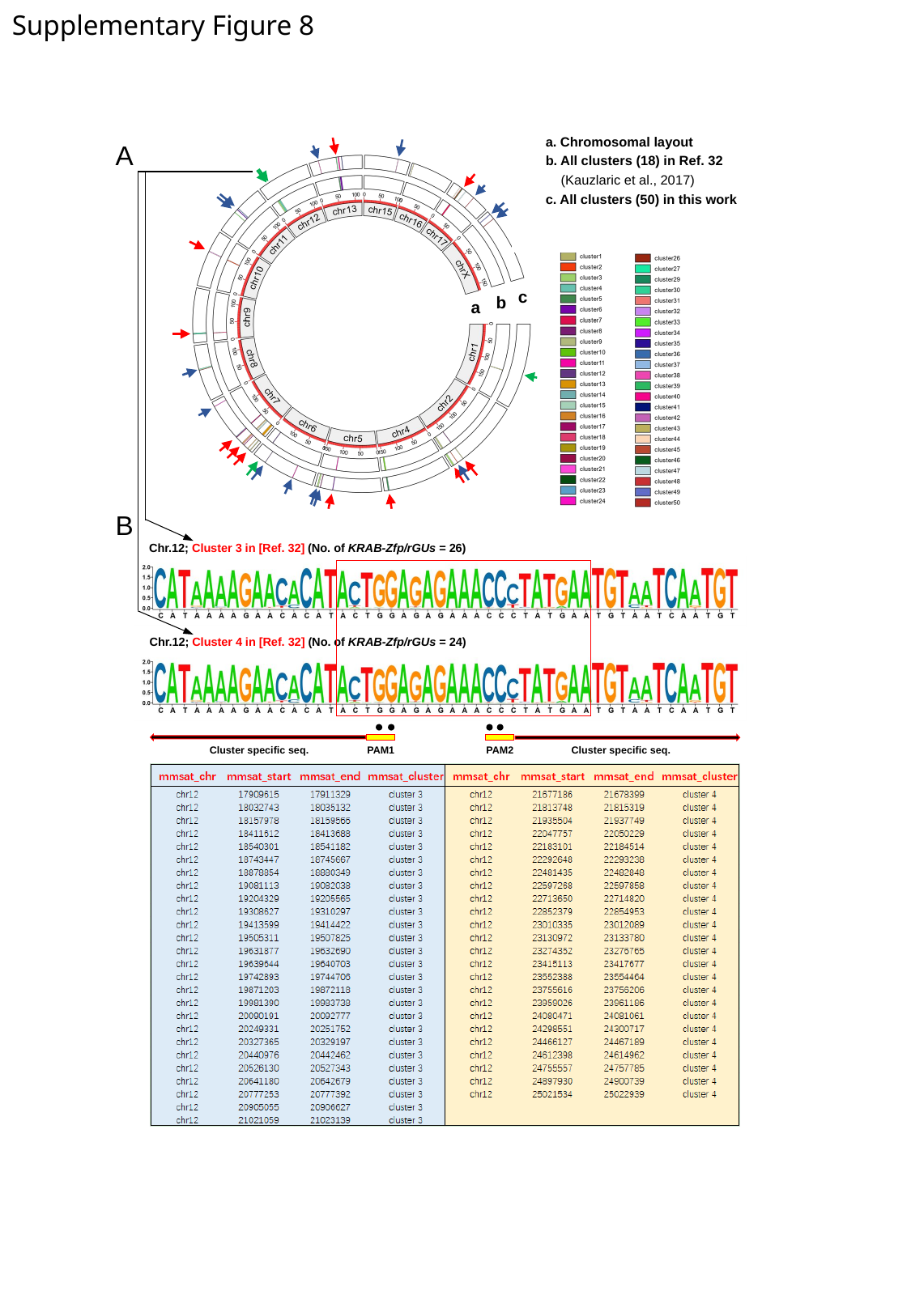

Supplementary Figure 8
a. Chromosomal layout
b. All clusters (18) in Ref. 32
 (Kauzlaric et al., 2017)
c. All clusters (50) in this work
A
c
b
a
B
Chr.12; Cluster 3 in [Ref. 32] (No. of KRAB-Zfp/rGUs = 26)
Chr.12; Cluster 4 in [Ref. 32] (No. of KRAB-Zfp/rGUs = 24)
Cluster specific seq.
PAM1
PAM2
Cluster specific seq.
